# Exploring Potential Achievements and Barriers to Provide Homecare for Women with Preeclampsia: A Qualitative Study

**DOI:** 10.30476/IJCBNM.2021.89368.1605

**Published:** 2022-01

**Authors:** Fatemeh Mohammadi, Shahnaz Kohan, Mohammadhossein Yarmohammadian, Mitra Savabi-Esfahani, Zahra Rastegari

**Affiliations:** 1 Nursing and Midwifery Care Research Center, School of Nursing and Midwifery, Isfahan University of Medical Sciences, Isfahan, Iran; 2 Reproductive Sciences and Sexual Health Research Center, School of Nursing and Midwifery, Isfahan University of Medical Sciences, Isfahan, Iran; 3 Health Management and Economic Research Center (HMERC), School of Medical Management and Information Sciences, Isfahan University of Medical Sciences, Isfahan, Iran; 4 Department of Midwifery, School of Nursing and Midwifery, Shiraz University of Medical Sciences, Shiraz, Iran

**Keywords:** Achievement, Barriers, Homecare, Iran, Preeclampsia, Qualitative study

## Abstract

**Background:**

Preeclampsia (PE) is one of the leading causes of mortality and complications during pregnancy. It seems that usual prenatal care is not enough for these patients. They require more assistance, support, and guidance from health professionals, and home care is an effective strategy in this regard. Also, Iran has no official or compiled program for home care in high-risk pregnancy. This study was designed to explore the potential achievements and barriers of home care for mothers with PE.

**Methods:**

In this qualitative study with conventional content analysis, twenty-eight participants (mothers with PE, maternal health policy-makers, and health care providers) were selected through purposeful sampling with maximum variation. Data were collected through semi-structured interviews until saturation was achieved. Simultaneously, data analysis was performed using MXQDA software. Finally, the main categories were extracted.

**Results:**

Seven main categories were extracted. Three main categories for the potential achievement included “family involvement in maternal care”, “holistic maternal health promotion”, and “improving utility of services”. The other four categories emerged for barriers included: “more willingness to provide in-hospital medical care”, “clients’ concerns about cultural issues”, “providers` unwillingness to delivery home care”, and “insufficiency of infrastructures for home care”.

**Conclusion:**

Paying attention to home care advantages, based on the socio-cultural context of the community, making effort to remove the barriers, and organizing home care infrastructures contribute to improvement in the quality of care in women with PE.

## INTRODUCTION

The maternal mortality is an indicator for women’s health condition in society. Therefore, the promotion of pregnant women’s health, as one of the priorities and the major concerns related to this issue, has atteracted the global attention. ^1^ Pregnancy-induced hypertension (PIH) and preeclampsia (PE) cause high-risk pregnancy that can lead to maternal death and morbidities. ^2^ The most important actions to protect these mothers’ health are providing high quality, effective, and efficient prenatal care. Especially, providing a part of pregnancy-related care at home to improve prenatal outcome and reduce complications is essential. ^3^ Since home care has been considered as a solution to reduce mortality and morbidity caused by high-risk pregnancies, ^4^ in recent years, providing preinatal home care services has grown rapidly in many developed countries, as a health promotion strategy. ^5^

Evidence shows that a home care program can have many benefits for the community and the health system, such as prevention of excess hospitalization, and reinforcement of interaction between health care providers and the community. ^6^ Also, it reduces the number of outpatient visits and health costs. ^7 , 8^ Eventually, it will lead to a positive return on investment to the health system. ^9^ In the same way, the antenatal home care service for pregnant women has many advantages for the mother, infants’ family, and health system. Decrease in the preterm labor and the number of infants’ hospitalization days in newborn intensive care unit (NICU) diminish financial burden imposed on the health system due to premature infants and neonatal mortality. ^10 - 12^ Enhancing family involvement in the caring of mothers declines the maternal stress and provides social support as well as appropriate and timely prenatal care for them. ^13^

A review of the literature revealed conflicting results from studies on home care delivery. However, a systematic review reported that a large number of studies showed that this intervention had no effect on pregnancy outcomes and child health. ^14^ Another study showed that the home visit program for the general population of pregnant women was successful. This intervention has reduced low birth weight and preterm delivery in most women, especially black women. ^15^

Also, there are other studies that report many challenges to providing home care services. Problems related to the socio-cultural context, establishment of communication and trust with family and clients, lack of infrastructure, understanding the language, absence of legal support, and ignoring ethical issues are among these cases. ^16 - 18^

Given the evidence, planning and providing home care seems to be necessary to improve the maternal and neonatal outcomes of pregnancy, but there is no home care program for pregnant women in the existing health system of Iran. Furthurmore, inconsistency in the findings of studies, as well as the impact of cultural, economic and social factors on home care programs designed for the community, makes it necessary to conduct a study before designing any program. Therefore, for research in areas where there is little information available, the use of qualitative research methods is recommended. ^19^ This study was conducted to explore the potential achievement and barriers of home care among women with PE using a qualitative method.

## METHODS

This study was a qualitative conventional content analysis conducted from 2018 to 2019. The study settings were health care centers and two public hospitals in Isfahan, Beheheshti and Alzahra. These two hospitals are important centers for referral and treatment of high-risk pregnant mothers in Isfahan. Participants consisted of midwives, obstetricians, maternal health care providers, and policymakers. The inclusion criteria for service providers and policy makers was having more than one year of work experience. Also, pregnant women who experienced PE and were able to communicate and speak in Persian and were willing to participate in the study were included. For all groups who participated in the study, unwillingness to continue cooperation was the exclusion criterion. The sampling method used was purposive sampling with maximum variation (age, gravidity, occupation, and educational level, hospitalization history, and professional position for providers and policy makers).

Semi-structured interview was used to collect the data. Semi-structured interviews are an effective method for data collection when the researcher tends to: (1) collect qualitative, open-ended data; (2) explore the participant’s thoughts, feelings, and beliefs about a particular topic; and (3) develop deeply into personal and sometimes sensitive issues. ^20^

Selection of the participants was based on their experiences about our research question or key concepts. Prior to the interview, the participants who were eligible for the study were contacted, and if they agreed to participate in the study, the place and time of interview were determined based on their willingness, for example at the clinic, office, or their house. An interview guide was designed using the literature review and opinions of experts that did the interviews. Some of our interview guide questions included: “What issues have you encountered since you have noticed your blood pressure is high? Please explain about it? Which type of care have you received so far? What do you think about receiving care at home? What are pros and cons of home care service upon your opinion?”

The interview with prenatal service providers began with a general question: “According to your experiences, would it be possible to take care of these mothers at home? What do you think will be the benefits of providing home care, for patients and the health system? What problems will we face if we want to provide home care services?” The questions the policymakers were asked included: “What benefits and disadvantages do you see in home care planning for mothers with PE? What changes have to be made in the health system or other organizations in your idea to provide care at home?”

The interviews lasted between 45 and 90 minutes, and continued until data saturation, when no new data were extracted. We recorded the interviews with audio tape and listened to them several times and transcribed them. The transcription was done in Microsoft word; then, they were imported in MXQDA 2018 to perform the data analysis, and the data were simultaneously analyzed. The text was analyzed using conventional content analysis as an inductive approach proposed by Graneheim and Lundman. ^21^ According to this method, the code scheme is directly extracted from the data. The codes were then extracted after reading the texts word by word and organized by their similarity to form the subcategories. The analysis continued and similar subcategories were grouped until the main categories emerged. 

To confirm the trustworthiness of the data, we evaluated four criteria proposed by Lincoln and Guba. ^22^ Prolonged engagement in data gathering, maximum variation, peer checking, and member checking were used to confirm the data credibility. Also, the experiences of the second and third authors with this method improved the data credibility. Confirmability was also improved by performing the full description of the participants in terms of characters, culture, context, and process of the analysis, using purposive sampling and observing maximum variation. External auditing by two qualitative researchers was performed to provide dependability. For transferability, the extracted categories were given to three individuals with the characteristics of participants who were not involved in the study, and their judgment was sought about the similarity between the results of the study and their experiences to achieve a good agreement.

### Ethical Considerations

Ethical approval was received for this study from the Ethics Committee of Isfahan University of Medical Sciences in Iran with the ethical code of IR.MUI.REC.1395.3.956. The participants were informed about the purpose of the study and signed the written consent. They were assured that their information would be kept confidential, and the interview audio files would be kept in a safe place. In addition, they were allowed to withdraw from the study at any time, and this did not affect the process of providing care to mothers. 

## RESULTS

This study was conducted on 17 maternal health services providers (eight midwives who worked in the health centers and hospitals with more than 10 years of professional experience, two midwifery instructors, two maternal health policymakers, and five obstetricians and reproductive health professionals) and 11 mothers who had experienced PE (Tables 1 and 2). Via content analysis of the interviews, seven main categories were extracted to explain the potential achievement and barriers of home care for pregnant women with PE. Three main categories about home care program “Potential achievements” and four main categories as “home care program barriers” are displayed in Table 3.

**Table 1:** Characteristics of pregnant women with preeclampsia in the study

Hospitalization History	Pregnancy Status During Interview	Number of Pregnancies	Occupation	Education	Age (Year)	Participant Number
Yes	34 Weeks	1	Housewife	Under Diploma	32	P10
No	32 Weeks	2	Housewife	Diploma	40	P11
Yes	31 Weeks	2	Housewife	Diploma	39	P14
Yes	30 Weeks	2	Housewife	Under Diploma	30	P15
No	31 Weeks	1	Employee	Bachelors’Degree	28	P16
Yes	28 Weeks	3	Housewife	High School	31	P23
Yes	35 Weeks	2	Housewife	Bachelors’Degree	33	P25
Yes	3 Days after Term Delivery	2	Housewife	Diploma	33	P18
No	2 Days after Term Delivery	2	Employee	Bachelors’Degree	35	P19
Yes	4 Days after Term Delivery	1	Housewife	Diploma	29	P22
Yes	34 Weeks	1	Housewife	Bachelors’Degree	36	P26

**Table 2:** Characteristics of the health providers and policymakers in the study

Duration of Work Experience(Years)	Professional Position	Education level	Participant Number
18	Faculty Member	Perinatologist	P27
30	Head of Department in Maternity Hospital	Ms in Midwifery	P6
18	Head of Department in Maternal Emergency	Ms in Midwifery	P2
21	Manager of Maternal Health	PhD in midwifery	P3
20	Chief of the Childbirth Specialized Hospital	Bs in Midwifery	P4
10	Faculty Member	Perinatologist	P13
12	Midwifery Staff in the Hospital	Ms in Midwifery	P7
22	Supervisor of HIgh-risk Mothers in Hospital	Bs in Midwifery	P5
23	Supervisor in Hospital	Bs in Midwifery	P9
10	Faculty member	Perinatologist	P12
21	Manager of Maternal Health	Ms in Midwifery	P21
10	Staff of Health Care Center	Bs in Midwifery	P24
21	Staff of Health Care Center	Bs in Midwifery	P28
23	Staff of maternal clinic of hospital	Bs in Midwifery	P8
30	Faculty Member	Ms in midwifery	P1
23	Staff of Health Care Center	Bs in Midwifery	P20
15	Faculty Member	Perinatologist	P17

**Table 3:** Categories and sub-categories of “potential achievements” and “barriers” of home care program for women with Preeclampsia

Sub categories	Categories
**Home care program potential achievements**
1-a-Family self-efficacy in care of mother	1-Family involvement in maternal care
1-b-Enhancing relationship between mother, family and care provider	
2-a-Improving physical health	2-Holistic maternal health promotion
2-b- Promoting mental health	
2-c- Providing social supports	
3-a-Reduced unnecessary visit in health centers	3-Improving utility of services
3-b-Equity in the provision of services and continuity of care	
**The barriers of home care program**	
4-a-Worry about not being able to follow the patient at home	4-More willingness to provide in-hospital medical care
4-b-Treatment-oriented care	
5-a-Fear of letting unfamiliar individuals inside the home	5-Clients` concerns about cultural issues
5-b-Concern about privacy disturbance	
6-a-Concerns about going to peoples’ home	6-Providers` unwillingness to delivery home care
6-b-Ethical and legal consideration in home care services	
7-a-Lack of inter-sectoral collaboration	7-Insufficiency of infrastructures for home care
7-b-Poor inter-professional cooperation	
7-c-Cost imposition	

### Home Care Potential Achievement

Home care potential achievement was one the findings that consisted of three categories: "family involvement in maternal care","holistic maternal health promotion”, and”improving utility of services“.

1-**Family involvement in maternal care:** In prenatal care, family members can play an important role in improving maternal and infant health; therefore, by providing home care, the family will become aware of the complications of pregnancy, and they will also be able to follow up the problems as much as possible. This category is formed from two subcategories, "family self-efficacy in the care of mother" and “enhancing relationship among mother, family and care provider".

1-a-Family self-efficacy in the care of mother: One of the benefits of providing home care for pregnant women with PE is family self-efficacy in caring of pregnant mothers because family participation in the process of caring is an inevitable part of this kind of care. The family gradually becomes aware of the needs and taking care of the pregnant woman, and they can make decisions to bring the pregnant woman to the health center at the appropriate time. A midwife said: *“When we go to the home of a pregnant woman with PE, the family also participates in the process of caring for the mother, and we can empower them to do so by training them. In my opinion, this will be very effective in improving care.”* (P6).

1-b-Enhancing the relationship among the mother, family and care provider: Improvement of interpersonal interactions between the family and health care providers is another achievement of home care. This achievement is acquired by spending sufficient time to communicate with the pregnant mother and the family during home care. In this regard, supervisor of the maternity department said: *“At the clinic, we usually do not see the pregnant mother’s family and do not know what her living conditions are like. In this case, for follow-ups and health and treatment orders, etc., only the mother addresses us. By going home, we communicate with their spouse, children, or those who live with them other.”* (P9).

2- **Holistic maternal health promotion:** The ultimate goal of home care, as community-based health care, is promoting all dimensions of maternal health and self-care such as "physical, mental, and social health of the mother".

2-a- Improving physical health: Most providers believed that one of the important and influential dimensions of home care is helping to improve the physical problems of the mothers identified in the health care centers. In this regard, one of the providers stated: *“By going to the home of the pregnant mother and checking the mother’s blood pressure and weight, many subsequent damages are prevented, like chronic blood pressure in future.”* (P6).

2-b- Promoting mental health: The analysis of the participants’ interview indicated that complications of pregnancy generate serious psychological responses; it can be prevented by delivering a comprehensive care at home to promote maternal mental health. A mother with PE said: *“I went to the hospital clinic, and I was very stressed when I was told that I had PE; I was so worried that I could not sleep at night. If one of the staff had talked to me more about this, I would have been less worried. Usually, midwives and doctors do not have the time to listen to us, so if someone comes home to care for us, this opportunity will be made.”* (P14).

2-c- Providing social supports: In addition to providing health services, the results of the data analysis indicated that some participants agreed that mothers also could receive more social support by being taken care of at home. One of the midwives emphasized *“As soon as the health visitor goes home and is faces with people, she listens to the mother’s sadness and concerns closely and is able to do something for them and can even link her to other supportive organization or person. This is an influencing factor in social support that she can handle her difficulties in this period.”* (P4)

3- **Improving the use of services:** Health care providers mentioned that providing home care is a key step in improving the mothers’ access to appropriate and qualified health care services. This category was formed from two sub-categories: “reduced unnecessary visit in health centers”, and “equity in the provision of services and continuity of care.”

3-a- Reduced unnecessary visit in health centers: The majority of health care providers stated that one of the problems in the current health care system was the overcrowding in healthcare centers, which causes problems for both the mother and the caregiver. In this regard, a midwife at the prenatal clinic said: *“health centers are very busy. By caring a pregnant mother at home, we can teach her how to monitor her blood pressure and weight, so she can identify when it is necessary to refer to the health center.”* (P2)

A mother said, *“We are waiting a long time for a visit at the clinic. It’s easier for us if a midwife comes to our home; I would not have to go to a clinic for checking the blood pressure. And the clinics will become more relaxed; they can only visit people with serious problems.”* (P 25)

3-b- Equity in the provision of services and continuity of care: Some health providers pointed out that preserving good health is the public right. One of the other benefits of provision of care at home is the development of equity in the community. A midwife said: *“Home care would be suitable for an individual in a low socio-economical condition who can’t access health care. It can even be used for families with a good financial situation in any socio- economic condition, to drop extra visit.”* (P7)

### The Barriers of Home Care Program

Analysis of the results showed four main categories for barriers of providing home care, including: “more willingness to provide in-hospital medical care”, “clients’ concerns about cultural issues”, “providers’ unwillingness to deliver home care”, and “insufficiency of infrastructures for home care.”

4-More willingness to provide in-hospital medical care: Fear and anxiety of the medical staff about maternal health, even in mild PE, have caused them to hospitalize more mothers to receive excess treatment. This main category was formed from two subcategories: “worry about not being able to follow the patient at home” and “treatment-oriented care”.

4-a-Worry about not being able to follow the patient at home: One of the main reasons for excessive hospitalization and refuseal to provide outpatient care or home care by health personnel is their concern about the worsening of the disease and lack of follow-up system for the mother. In this regard, a midwife said: *“When a mother with PE goes home, it’s all our concerns whether the patient might come back with an emergency status, so we prefer to admit the mother with the minimum problem in the hospital. Many times, we will force mothers to be hospitalized.”* (P2)

4-b- Treatment-oriented care: Most antenatal care clinics are run by physicians alone and have no connection to other parts of the health system. Therefore, in most cases, physicians use medical treatment instead of monitoring the patient on an outpatient basis or at home. A care provider said, *“When a mothervrefers to the clinic, the physicians are the only ones who decide on patient management and medical treatment.”* (P7)

5-Clients’ concerns about cultural issues: One of the remarkable findings of our study which hinders the provision of home care for mothers with PE was the lack of cultural acceptability of home care in our country. This category emerged from two sub-categories, including “fear of letting unfamiliar individuals inside home” and "concern about privacy disturbance".

5-a-Fear of letting unfamiliar individuals inside home: Many participants believed in the welfare of home care, but the reason for not accepting this program is lack of willingness in allowing the stranger to enter their home by the husband and family members. One of the mothers said: *“But I’m worried someone comes to my home. Since I often stay alone in my house, in the morning; I prefer to go to the health center near our house. Furthermore, my husband does not allow someone to come for home care.”* (P15)

5-b- Concern about privacy disturbance: Some mothers were reluctant to allow the staff enter their homes because they were afraid that their privacy would be compromised. A pregnant woman said: *“When a caregiver comes to my house, my neighbors inquire about it. An issue is that if you come to my home, the problems of my life may be disclosed.”* (P16)

6-Providers’ unwillingness about delivery of home care: Other obstacles to the implementation of the home care program were “providers’ concerns about going to peoples’ home” and “ethical and legal consideration in home care services”.

6-a- Concerns about attending peoples’ home: Most of the providers who participated in this study pointed out that safety and security at peoples’ home should be considered; in this regard, a midwife said: *“The health provider that attends someone’s home might be exposed to many threats, and so many caregivers don’t accept this program because of insecurity. We all know the conditions of the community; we have a lot of safety problems. Safety should be provided for the health provider in case he/she attends one’s home.”* (P5)

6-b – Ethical and legal consideration in home care services: The majority of the health care providers were concerned about the professional, ethical, and legal issues in providing home care while bearing in mind the limitations on their responsibility andlack of clear rules and regulations on their task and duties. A midwife stated: *“Caregivers were reluctant to go to people’s home because of the lack of certain protocols and deficits in law in support of them for home care in our health system. Conflict on the legal matter is one of the major challenges of home visitors.”* (P9)

7- Insufficiency of infrastructures for home care: Since the provision of home care is a new structure for our health system, this service requires setting up infrastructures interrelated to other social organizations. This category was formed from three subcategories:” lack of inter-sectoral collaboration”, “poor inter-professional cooperation”, and “imposing costs.”

7-a- Lack of inter-sectoral collaboration: According to the participants, another obstacle in implementing home care for pregnant mothers was lack of collaboration of other sectors with the health system. One of the maternal health officials in this regard said: *“I believe home care for high-risk pregnant mothers is a specialized technical service, and for placing this type of health services in the structure of health system, other organizations such as insurance and security agencies should be involved and collaborate.”* (P3)

7-b- Poor inter-professional cooperation: Analysis of the participants’ interview in this field indicated thatphysicians do not support home care; one of maternal health administrators stated: *“For implementing this kind of care, coordination between the health providers, physicians, and health care system must be done. This will take a long time because of the deficiencies in teamwork in our health system.”* (P4)

A physician said: *“In my opinion, doctors will never recommend home care for high-risk pregnant women, and they don’t take responsibility to do it. From another perspective, they may disagree with this due to the low income of physicians.”* (P12)

7-c- Cost imposition: Among the most important and challenging categories of providing care at home was its economic burden. Increasing family expenditures and lack of coverage by insurance and health system support impose extra costs.

Most of the pregnant women stated that they could not afford the potential cost of home care, and that receiving this care imposes additional costs on the family. A mother said: *“Many families may be willing to receive this care at home, but I personally prefer to go to health clinics because of its free charges. I know that my family cannot afford to pay for the home care.”* (P22)

One of the policymakers of the maternal health department said: *“Home care is costly for the Health Ministry, so families should pay for this; implementing this program is financially difficult, so the support of insurance organization is essential.”* (P3)

## DISCUSSION

This research explains the potential achievements and barriers of home care for pregnant women with PE that emerged as seven main categories. Findings of the potential achievements in this study include family involvement in maternal care, holistic maternal health promotion, and improvement in the use of services.

One of the most important potential achievements of home care from the the perspective of the majority of participants was involvement of the family in promoting the health of the mother. Similarly, other studies have shown that providing home care services with family involvement can improve maternal care. This way, the caregiver provides a description of the situation for the family as well as the spouse and educates them to get involved in the mother care. ^23^ In addition, the mother is encouraged to discuss her situation with her husband and family members and inform them about the problems and the ways to manage them. ^24^

Improving various aspects of maternal health is another potential gain of home care which was concluded from the present study. Evidence shows that in the health centers and clinics, as a result of the time limitation in visiting the pregnant mothers, the main complaint or problem is addressed and other aspects are not considered. Mental health of the pregnant women in most health centers is also neglected during prenatal care. ^25^ Therefore, home care can decrease maternal, emotional, and social problems in high-risk pregnancies. ^26^ In most studies, the provision of care at home increases self-esteem, reduces depression, and subsequently improves mental health and quality of life in pregnant mothers. ^27^

Results show that improving the use of services and equity in service delivery is also one of the benefits of home care for mothers. In line with our finding, a study revealed that home care should be considered as an effective approach to reducing inequality in the care of pregnant mothers. ^28^ A study also stated that constant prenatal care through the development of home care services could significantly reduce the burden of additional visits to the health care centers and subsequently improved the quality of maternity care. ^29^ Also, the World Health Organization recommends that a part of prenatal care is provided at home because it is one of the most effective interventions to reduce health inequities. ^30^

Also, our study shows four barriers to providing home care services for women with PE, more willingness to provide in-hospital medical care, clients’ concerns about cultural issues, providers’ unwillingness to deliver care at home, and insufficiency of infrastructures for home care. 

The findings of this study demonstrated more willingness of caregivers to provide in-hospital medical care (they worry about not being able to follow the patient at home and treatment-oriented care) for high-risk pregnant women in Iran which can be an obstacle to providing services to these women at home. Likewise, a study also stated that in Iran, even in low-risk pregnancies, unnecessary medical interventions are usually performed. ^31^

Mothers’ concerns about allowing the health providers to enter their home and disturbance of their privacy are cultural barriers to providing maternity care at home. Results of a research also mentioned the fear of families facing an unknown care as one of the barriers of using home care services by mothers. ^32^ In the same line, a study found that the mother-in-law played an important role in deciding whether to take prenatal care at home or not. ^33^ Different studies emphasized that cultural issues must be respected for better acceptability of services. A study indicated that socio-cultural factors of Iran’s society affected accessibility and utilization of reproductive health services. ^34^

On the other hand, health providers were concerned about the insecurity of attending people’s house and the legal aspects of providing home care. Another study showed that health professionals pointed to problems with workload, transportation issues, and dangers as barriers to postnatal home care. ^35^ For overcoming these problems, various measures must be taken to ensure security in home care. Some of these solutions should be provided by the employers, such as training the security considerations to the personnel, establishing a supervision system, coordinating with the police or security guard, and providing facilities for declaring a security emergency. In addition, caregivers should also observe points such as the initial visit to the house with a companion, coordination of the time of arrival and departure with the supervisors, and case reports such as stealing their equipment or car at the place of visit, etc. ^36^

Lack of intra-sectoral and inter-professional collaboration and imposing costs which results from home care services are the other barriers revealed in the present study. The result of a study also revealed that the integration of home care services in the existing services, creation of inter-organizational cooperation to use the capacities of governmental agencies and charities, and formation of specialized home care teams from different health-related professions were essential. ^37^ However, participants in our study believed that providing home care services would impose costs on the family and the health system. One study showed that providing home care services reduced the number of hospitalizations, and, in the long run, it reduced the costs of caring for the patient and treating the disease . Another research shows that reducing the use of home care services increases the number of referrals to the emergency room, hospitalization, and complications in patients and imposes a heavier burden on the health system in terms of cost. ^38^ The reason for the discrepancy observed in the results of the present study with the other two studies is that in Iran, services in most hospitals are covered by insurance, but services such as home care, which is currently provided for other diseases, are not covered by insurance, and it imposes more costs on the patient and family.

The strengths of this study were its qualitative design and data collection which were through semi-structured interviews. Qualitative approach could help us view the data more extensively and deeply about the conditions affecting home care for PE that was reported for the first time. In addition to pregnant women with PE, policymakers, physicians, and prenatal care providers were also interviewed as the pioneers of health care providers in Iran. The limitation of this study was that home care is not provided for pregnant women in Iran, so the participants did not have a complete and vivid experience about it.

## CONCLUSION

Home care of women with high-risk pregnancies, especially PE, has some benefits, such as family involvement in maternal care, and holistic maternal health promotion, and improvement of the utility of services. It is necessary to provide maternal health care based on the socio-cultural context of the community through efforts to overcome the barriers and improve the existing infrastructure. Accordingly, planning and providing community-based home care can be effectively managed by mothers with PE in Iran. In addition, in the case of PE, which is a major concern for pregnant mothers, the emphasis should be placed on changing the midwives and physicians’ approach to reduce excessive hospitalization/visiting and on further follow-ups with the home care.

## ACKNOWLEDGEMENT

We would like to thank the vice-chancellor for research of Isfahan University of Medical Sciences for their support and also participants for taking part in this research. This study is part of a PhD dissertation with the research code of 395956, conducted by Zahra Rastegari. We thank the vice-chancellor for research of Isfahan University of Medical Sciences for the support, and also, participants for taking part in this research

**Conflict of Interest:** none declared.
